# Segmentation of biomedical images using active contour model with robust image feature and shape prior

**DOI:** 10.1002/cnm.2600

**Published:** 2013-10-28

**Authors:** Si Yong Yeo, Xianghua Xie, Igor Sazonov, Perumal Nithiarasu

**Affiliations:** 1 Institute of High Performance Computing1 Fusionopolis Way, Singapore 138632, Singapore; 2 College of Engineering, Swansea UniversitySingleton Park, Swansea SA2 8PP, UK; 3 Department of Computer Science, Swansea UniversitySingleton Park, Swansea SA2 8PP, UK

**Keywords:** image segmentation, active contour, level set model, shape distribution, Bayesian inference

## Abstract

In this article, a new level set model is proposed for the segmentation of biomedical images. The image energy of the proposed model is derived from a robust image gradient feature which gives the active contour a global representation of the geometric configuration, making it more robust in dealing with image noise, weak edges, and initial configurations. Statistical shape information is incorporated using nonparametric shape density distribution, which allows the shape model to handle relatively large shape variations. The segmentation of various shapes from both synthetic and real images depict the robustness and efficiency of the proposed method. © 2013 The Authors. *International Journal for Numerical Methods in Biomedical Engineering* published by John Wiley & Sons, Ltd.

## 1. Introduction

Image segmentation involves the partitioning of an image such that objects of interest can be extracted from the image background. Shape segmentation is an important area in biomedical image analysis and has a wide range of uses such as tissue classification, virtual endoscopy, image-guided surgery, diagnosis, biomedical simulation, and image-based modeling. Geometric reconstruction from biomedical image volumes by manual labeling can be very tedious due to the sheer size of the image datasets, and the complexity and variability of the anatomic object shapes. Also, inter and intra variability of the geometries extracted manually by different users, and geometries extracted by the same user at different times, can be considerably large. It is therefore useful to design a robust algorithm for the automatic delineation of anatomic structures from images acquired from different imaging modalities such as magnetic resonance imaging, computed tomography imaging, and ultrasound imaging.

There have been multitude of approaches proposed for automatic object segmentation from biomedical images 1–16. Many of those work are specifically designed for particular applications, e.g., vascular structures 1,2,6,11,14, skeletal structures 3,7,15, airway 5, brain 9,16, and heart 12. However, automatic segmentation remains to be an intricate task. Some of the main challenges include the extraction of object boundaries or regions from images with noise and intensity inhomogeneity, which often exist in biomedical images due to factors such as sampling artifacts and bias field. Other factors such as weak object edges, low resolution, and spatial aliasing can also affect the accuracy and efficiency of the shape extraction process.

Active contours or deformable models provide an effective framework for object segmentation and has been widely used in biomedical image segmentation, as they can easily adapt to shape variations 17–21. Various types of useful information can also be incorporated to regularize the smoothness and shape of the contour. Recent advances in variational formulation, such as 22, provides new possibilities in numerical implementations. However, it is still a great challenge for active contour models to achieve strong invariance to initialization and robust convergence. This is particularly true when the active contour is applied on real image datasets consisting of intensity inhomogeneity and complex geometries. In the presence of artifacts, occlusions or large amount of noise, it is difficult for purely image-based models to extract image objects accurately. In such cases, prior knowledge of shape information can be very useful as it provides a constraint to the deformation of the contour such that the model favors similar shapes represented in the training set.

One of the earliest approaches in modeling shape information uses an explicit representation of the shapes. In 23, the training shapes are represented using landmark or control points, and principal component analysis (PCA) is used to model the variability of the training set. The algorithm is based on a parametric point distribution model that uses linear combinations of the eigenvectors to represent variations from the mean shape. The shapes are aligned using an iterative technique called the procrustes analysis 24, such that the shape model is more robust to rigid transformations such as translation, rotation, and scaling. The use of landmark points, however, has a drawback as the accuracy of the shape analysis depends on the quality of the landmarks. In addition, such shape models require the parameterization of the active contours. Recently, various groups 25–28 have incorporated shape prior information into the level set framework. In 25, shapes are represented using signed distance functions, and PCA is applied to the training shapes. The prior information is then incorporated into a geodesic active contour 29 to attract the level set function toward similar shapes represented in the shape distribution. The shape model is composed of the mean shape and a weighted sum of the principal modes of variation. In 30, PCA is applied to the space of signed distance functions, and the parameters of the principal eigenmodes are optimized efficiently. The signed distance functions are more robust to slight misalignments of the training shapes than parametric contours. However, the space of signed distance function is nonlinear, and the shape representations using linear combination of eigenmodes do not, in general, correspond to a signed distance function. In 31, the shape information is imposed onto the contour extracted from the level set function at each iteration. The shape prior, therefore, acts on the contour and has difficulties in modelling topological changes.

In general, many of the shape models are based on statistical assumptions that the training shapes are distributed according to a Gaussian distribution. This can easily restrict the range of applications as real world objects can often exhibit complex shape variations, and the projection from 3D object to 2D image can be nonlinear. In 27,28,32, the kernel density estimation (KDE), which is a nonparametric technique for the estimation of probability distribution functions, is applied to the space of shapes to model the shape distribution. This allows the model to handle a relatively large variation of shapes.

In the present work, we propose a variational level set model for shape segmentation using Bayesian inference. The proposed model uses an image-based energy and shape-based energy to attract the active contour toward the object shape. Image intensity and color or their local distributions has been commonly used to derive the image energy, such as in 27,32. Texture information can also be used; however, they may form a large dimensional feature space, which can be difficult to formulate in the level set framework without cascading the feature vectors that may reduce its discriminability. Image intensity gradient is sensitive to image noise and weak edges as it uses local image information, and region-based models are often affected by intensity variations. In the present work, the image-based energy is therefore derived from the global interaction of image intensity gradient vectors. This gradient vector interaction field is also known as the geometric potential field, and we have shown in 21 that its vector form can increase the robustness and efficiency of the active contours in handling image noise, challenging initialization, weak edge, and even broken object boundaries. Its scalar form is used in the proposed model as an image feature to indicate the presence of object boundaries as described in 33. Its characteristics are fundamentally different from image intensity or image intensity gradient, as it exhibits a coherent and global geometric configuration of the image objects. The shape-based energy is incorporated into the segmentation model using nonparametric shape distribution 27. The use of the nonparametric technique of KDE allows the shape prior to model arbitrary shape distributions and can therefore handle large shape variations in the training set. The proposed model, which consists of the image-based and shape-based energy, allows the active contour to efficiently handle feature inhomogeneity, occlusion, image noise, and weak object edges.

## 2. Methods

In this section, the formulation of the proposed level set-based segmentation model is presented. The proposed model consists of an image attraction force, which propagates contours toward object boundaries, and a global shape force, which deforms the model according to the shape distribution learned from a training set. The image attraction force is derived from the interaction of gradient vectors. It differs from conventional image intensity gradient-based methods, as it utilizes pixel interactions across the whole image domain. A shape distance is defined to measure the dissimilarity between shapes. The statistical shape information is incorporated into the model by using nonparametric shape density distribution 27,32 of the training shapes.

### 2.1 Bayesian formulation of segmentation model

In this section, the segmentation model is formulated using Bayesian inference, where the segmentation of an image represented by the image intensity *I* can be considered as maximizing the conditional probability given as

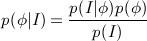
(1)
Here, *p*(*ϕ* | *I*) denotes the posterior probability, and *p*(*I* | *ϕ*) is called the likelihood that is the probability of *I* given the shape *ϕ*. The shape that maximizes the posterior probability distribution can be estimated using a maximum a posteriori approach:

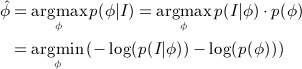
(2) because *p*(*I*) is independent of the shape *ϕ* and is constant for a given image. The maximum a posteriori estimation of the shape in Equation (2) that maximizes the posterior probability can also be achieved to minimize the following energy functional:

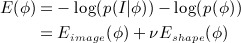
(3)
where *E*_*image*_(*ϕ*) represents the image-based term, *E*_*shape*_(*ϕ*) represents the shape prior, and *ν* is a constant that controls the influence of the shape-based energy. Note that maximizing the posterior probability in Equation (1) is equivalent to minimizing the negative log-likelihood, which is given as a sum of the energies *E*_*image*_(*ϕ*) and *E*_*shape*_(*ϕ*) in Equation (3). The minimization of the energy functional *E*(*ϕ*) can therefore be interpreted as a segmentation model that simultaneously maximizes the accuracy of the object boundaries located by the evolving shape, and the similarity of the evolving shape with respect to the shapes represented in the training set.

### 2.2 Image-based energy

The image-based term is used to propagate the model toward the feature of interest in the image and can be image intensity gradient-based or region-based. Image object edge-based approaches represent object boundaries using image intensity gradient, while region-based approaches use characteristics such as color and texture to define the region within an object. Image gradient-based methods can be very effective when object boundaries are well defined. As conventional edge-based methods 29,34 attract contours toward object boundaries using external energies derived from local image information, they are often affected by local minima such as image noise and have difficulties in dealing with weak object edges. The gradient vector flow model in 35 uses vector diffusion, which increases the attraction range and allows the model to handle boundary concavities. It, however, has convergence issues caused by saddle or stationary points in its force field 36,37. Region-based methods make use of regional statistics such as means and variances to derive the external energies or forces and are thus more robust to noise interference. However, as region-based models 17,18,38 are often based on the assumption that image objects consist of distinct regional statistics, they cannot deal with intensity inhomogeneity in images. In 21, we derived a new image attraction force on the basis of hypothesized gradient vector interactions for contour evolution. Here, we define an image attraction feature on the basis of the gradient vector interactions and formulate the image-based energy in a variational framework such that statistical prior information can be conveniently incorporated into the model. The new image-based energy can thus be written as:

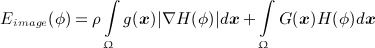
(4)
where *ρ* is a constant parameter that controls the smoothness of the contour, *g*(***x***) = 1 / (1 + | ∇ *I* |, and *H* is the Heaviside function. *G*(***x***) represents the gradient vector interaction field given as:

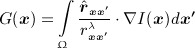
(5)
where 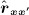 is the unit vector from pixel location ***x*** to ***x* ′** and *r*_***xx* ′**_ is the distance between the pixels, and *λ* is a constant, which coincides with the dimension of the image data (i.e., *λ* = 2 for 2D image). Note that the gradient vector interaction field in Equation (5) can also be expressed as a vector convolution defined as:

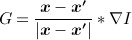
(6)
The first term in Equation (4) induces the segmentation model to favor minimal length, while the second term attracts the active contour toward object boundaries.

Although the gradient vector interaction field *G*(***x***) is derived from image intensity gradients, it utilizes image pixels or voxels across the whole image domain. As shown in Equation (5), the magnitude of the image force at each pixel ***x*** is based on the relative position and orientation of ***x*** with other pixels ***x* ′**. The proposed image feature, therefore, utilizes pixel interactions across the whole image and thus gives a global representation of the geometric configuration. This provides the active contour with a high invariance to initializations and a large attraction range. It also increases the robustness of the active contour against image noise. The correlation between image pixel locations gives *G*(***x***) its bidirectionality, and allows the active contour to handle arbitrary cross-boundary initializations and weak edges in the image.

### 2.3 Shape-based energy

**Figure 1:**
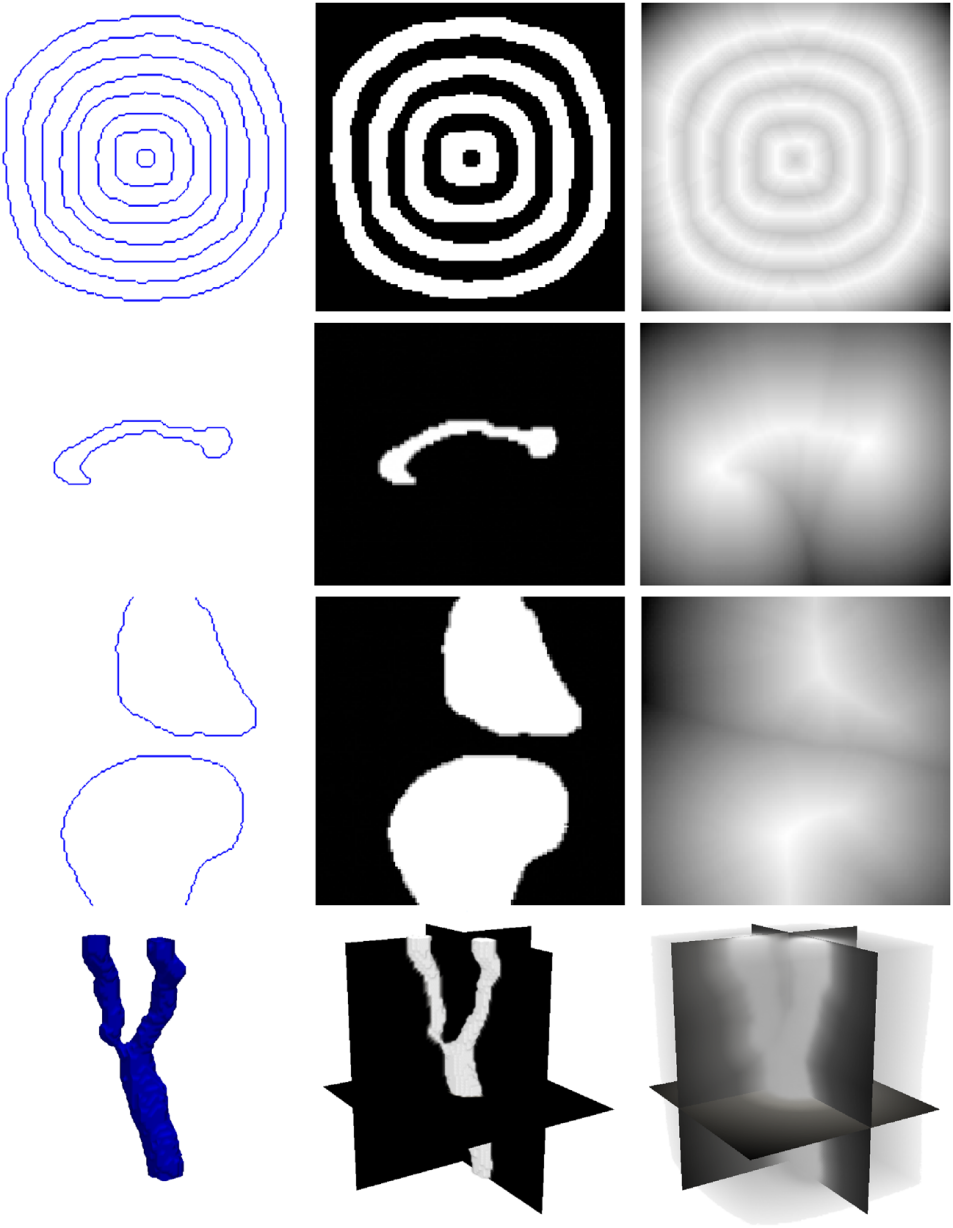
Various shape representations: (from left to right, top to bottom) contour or surface, binary image, and signed distance function representations of shapes for annulus-like objects, corpus callosum, knee, and carotid respectively.

In this section, a nonparametric technique 27 is used to generate a statistical shape distance measure for level set-based shape representations. The signed distance function, which can be conveniently derived from the level set function, is used to represent a shape. Figure 1 depicts the various representations of shapes. As shown in the figure, the signed distance function exhibits spatial correlation between the pixels and the object boundaries and can thus be effectively used for shape representations.

In order to derive a shape prior, a distance or dissimilarity measure for two shapes has to be defined. Given a set of training shapes {*ϕ*_*i*_}_*i* = 1 … *N*_, the shape energy functional based on the shape distance measure between the evolving shape and the training shapes *ϕ*_*i*_ can be defined as:

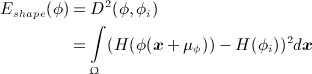
(7)
where *μ*_*ϕ*_ denotes the center of gravity of the shape *ϕ* and can be defined as 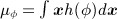, where 
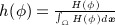
. Note that the training shapes *ϕ*_*i*_ are assumed to be aligned with respect to their center of gravity. The intrinsic alignment in the shape distance provides a dissimilarity measure, which is invariant to the location of the shape *ϕ*.

The nonparametric technique of KDE can then be used to model the statistical shape distribution. Here, the shape energy functional is defined on the basis of a probability density on the space of signed distance functions by integrating the shape distance (7) in KDE as

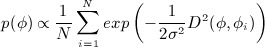
(8) where *σ* is the kernel width and can be set on the basis of the mean nearest-neighbor distance. The shape prior is invariant to the translation of the shape *ϕ*. Intrinsic alignments with respect to scale and rotation can also be incorporated in the model 27.

### 2.4 Variational level set segmentation model with global shape prior

The minimization of the energy functional in Equation (3) generates a segmentation model, which attracts the active contour toward image object boundaries and similar shapes in the training set. As the energy functional in Equation (3) is in the form of 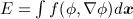, the Euler-Lagrange equation can be defined as:

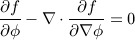
(9) The gradient descent with respect to the shape *ϕ* can therefore be used to minimize the energy functional in Equation (3) and is derived using calculus of variation as:

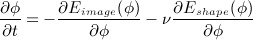
(10) The image-based gradient flow is given as (Equation (4)):

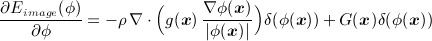
(11) where *δ* is the Dirac delta function. The shape gradient flow is defined as (Equation (7)):

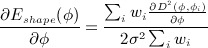
(12) which induces a shape force in the direction of each training shape *ϕ*_*i*_ weighted by the factor:

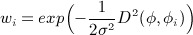
(13) The shape derivative with respect to *ϕ* is given as (Equation (7)):

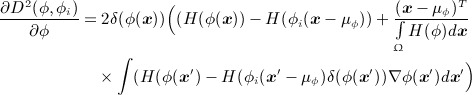
(14)

The variational segmentation model therefore maximizes the alignment between the active contour and the image object boundaries, and the similarity of the evolving shape with respect to the shapes represented using the statistical shape distribution.

### 2.5 Implementation details

The image object boundary representation used in the derivation of *G*(***x***) can be computed using central differences, or standard edge detection methods such as the Sobel filter. Some effects caused by spurious edges can be removed by not considering pixels with very small edge magnitude, i.e., 5 – 10% of the maximum magnitude. *G*(***x***) is computed efficiently as a vector convolution using FFT.

The Heaviside function *H* in Equation (4) is approximated by the regularized function *H*_*ε*_ defined in 17 as:

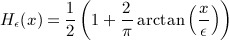
(15) and the Dirac delta function *δ* in Equation (7) is approximated by the derivative of *H*_*ε*_ as:

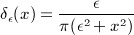
(16) where *ε* is an arbitrary small constant, i.e., *ε* = 1.0. The finite difference method is used to approximate the derivatives, and the narrow band approach 39 which considers only a narrow band of pixels around the level set interface is used to reduce the computational cost in updating the level set function.

The training set is generated or extracted manually in the form of binary images, and the signed distance functions, which represent the shapes, are computed using an efficient distance transform algorithm 40.

The curvature weighting parameter *ρ* in Equation (4) can be set to a small value such as *ρ* = 1.0 or set as 0, as the shape prior can effectively smooth out noise interference and regularize the shape of the contour. The weighting parameter *ν* in Equation (3) is used to balance the effects of the image and shape-based energy functionals on the segmentation process. The choice of *ν* depends on the specific application and the complexity of the images, and it is often required to tune this parameter for efficient segmentation 41,27,28. A *ν* value that is too small may cause the shape prior to have little effect on shape regularization, and a *ν* value that is too large may cause the global shape force to be too dominant and overwhelm local shape features. Here, the magnitude of the gradient vector interaction field *G*(***x***) is used as a guide to choose an appropriate value for *ν*, because it is the dominant component in the image-based gradient flow given in Equation (11). In particular, | *G* | is usually much larger than the magnitude of the shape gradient flow; therefore, *ν* is set to a considerably large value to balance the image-based and shape-based energy functionals. This can be done by setting *ν* to a fraction of the ratio of | *G* | _*max*_ to the maximum of the magnitude of the shape gradient flow at the initial configuration. It can be noted that once the appropriate *ν* values are chosen, they can be used to efficiently segment similar object shapes from a wide range of images of the same modality.

## 3. Results and Discussion

In this section, we show that the proposed method can be applied to efficiently segment image objects. The proposed method was compared against the Chan-Vese region-based model 17, and the Chan-Vese model with shape prior 27 in which the proposed shape-based energy is incorporated to the Chan-Vese model, on both synthetic and real images. In the experiments, a range of values for the parameters of the Chan-Vese model 17 and the Chan-Vese model with shape prior 27 are used, and the best outcomes for each model are selected to ensure a fair comparison.

### 3.1 Synthetic images

**Figure 2:**
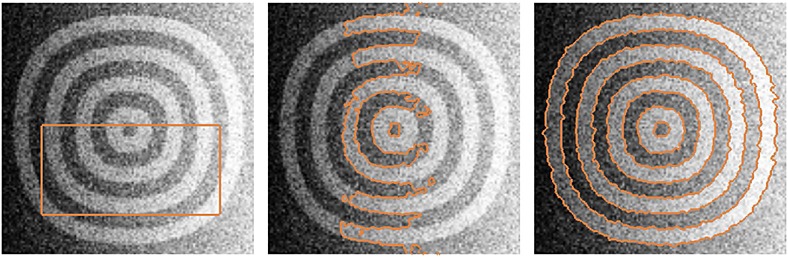
Segmentation of annulus-like shapes from noisy image: (from left to right) initial contour, region-based energy 17, gradient vector interaction-based energy.

Figure 2 depicts the segmentation of multiple annulus-like objects from an image with 40% noise and intensity variation. It is shown that the image-based energy derived from the global interactions of gradient vectors is robust to image noise and allows the active contour to extract the shapes accurately with an arbitrary cross-boundary initialization. Although the Chan-Vese model can handle the image noise, it cannot deal with the inhomogeneous intensity as shown in the figure.

**Figure 3:**
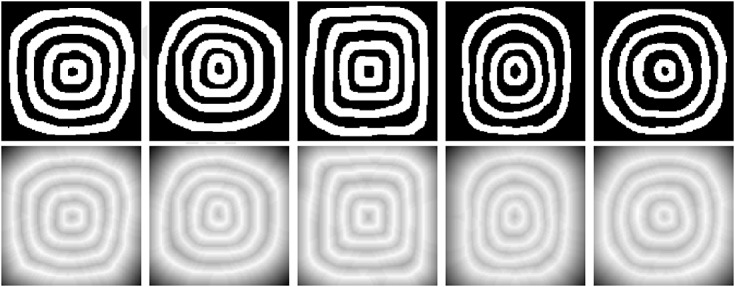
Training shapes of multiple annulus-like objects: (top row) binary images and (bottom row) corresponding signed distance functions.

Next, we consider a training set of 20 images with annulus-like objects of considerable shape variations, which we will use to derive the shape prior. Figure 3 depicts 5 of the shapes of the multiple annulus-like objects in the training set. The top row of Figure 3 shows the binary images, and the bottom row of Figure 3 shows the signed distance function representation of the training shapes.

**Figure 4:**
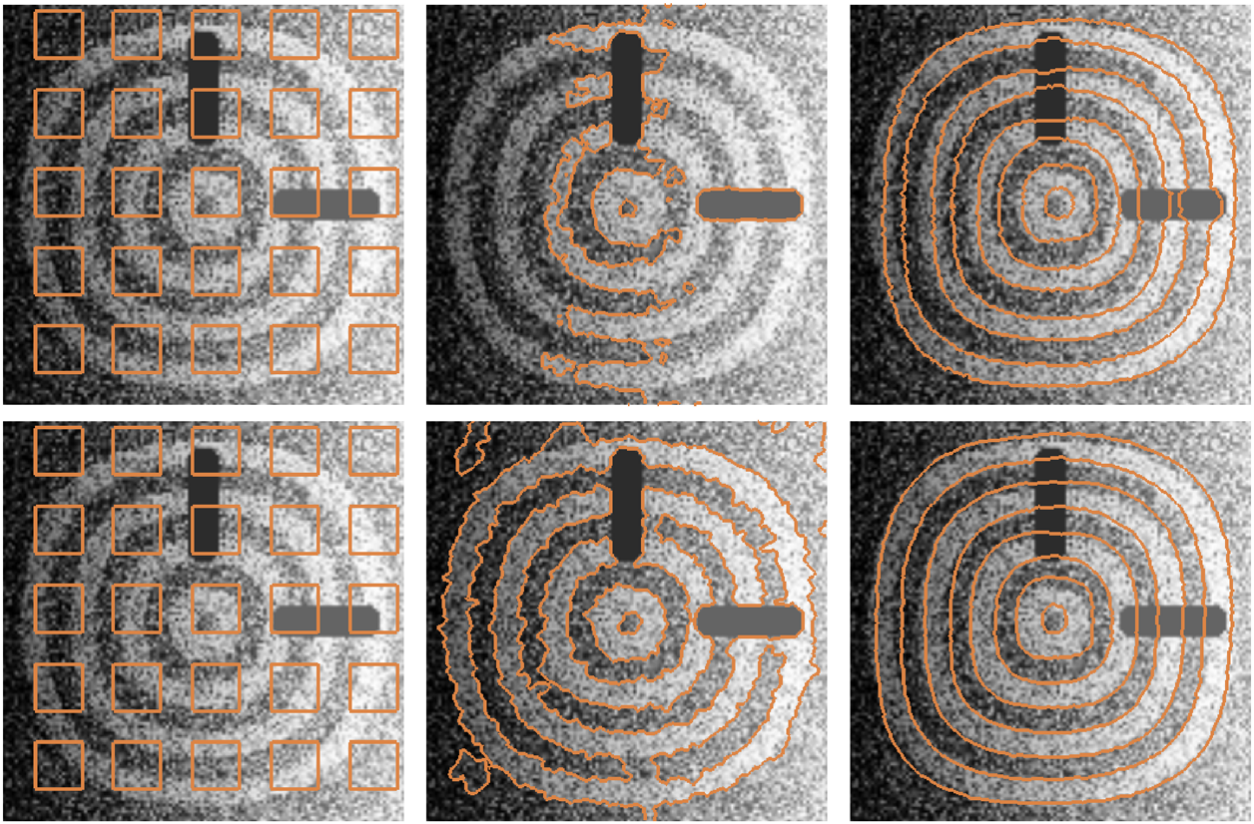
Segmentation of annulus-like shapes from occluded and noisy image: (top row, from left to right) initial contour, Chan-Vese model, Chan-Vese model with shape prior, (bottom row, from left to right) initial contour, gradient vector interaction-based energy, gradient vector interaction, and shape-based energy.

The shape prior is incorporated to the active contour models to extract the shapes from a noisy image (i.e., 70% of the pixels replaced by Gaussian noise), occlusions and intensity variation as shown in Figure 4. The top row of Figure 4 shows the segmentation of the annulus-like objects using Chan-Vese model and Chan-Vese model with shape-based energy. The bottom row of the figure shows the segmentation of the shapes using the proposed active contour with gradient vector interaction-based energy, and gradient vector interaction-based and shape-based energies. As shown in the figure, the shape force overwhelmed the region-based force in the Chan-Vese model 17, and did not locate the boundaries of the objects accurately. In contrast, the proposed active contour with shape prior extracted the shapes efficiently.

**Figure 5:**
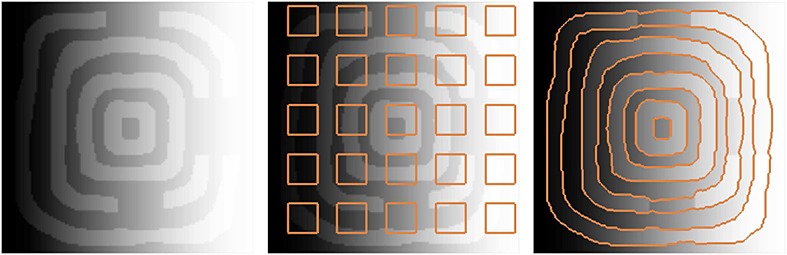
Segmentation of annulus-like shapes using gradient vector interaction-based and shape-based energies (from left to right) input image, initial contour, and converged model.

Figure 5 shows another example in which the proposed model with shape prior is applied to segment the annulus-like shapes with parts of the objects removed from the image. As shown in the figure, the gradient vector interaction-based energy allows the active contour to locate the object boundaries, while the global shape-based energy draws the active contour toward similar annulus-like shapes to segment the image objects effectively.

### 3.2 Real images

The proposed level set segmentation with shape prior is also applied to real images. In particular, it is shown that the statistical shape prior can be used to increase the robustness of the deformable model in the segmentation of biomedical structures such as the corpus callosum, knee joint, and carotid geometries from image datasets. In the various applications, the training shapes are manually segmented to model the shape distribution. The shape priors are then used in the segmentation of the biomedical structures from images of which the shapes are not included in the training set. As the shape prior information is incorporated using nonparametric shape distribution, the model can handle a relatively large amount of shape variability in the training set. The image-based and shape-based energies can therefore attract the contours toward image object boundaries and similar shapes represented in the shape distribution to minimize inter-operator variability.

**Figure 6:**
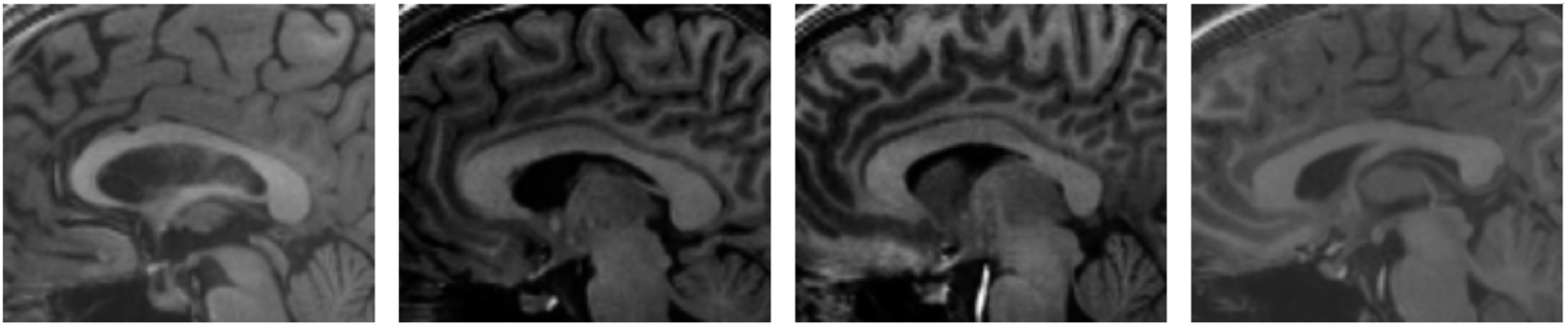
Various images of the brain: structures such as the white matter of the cortical regions and the fornix have similar intensity range and are connected to the corpus callosum.

Segmentation of the corpus callosum from magnetic resonance (MR) image can be challenging as the intensity range is similar to connecting structures such as the white matter of the cortical regions and fornix (see Figure 6). Therefore, segmentation models that use only image information may often include other white matter regions because of diffused object boundaries and similar region characteristics in the image. By incorporating prior shape information, the segmentation model can attract the model to similar shapes represented in the shape distribution to efficiently segment the structure from the image.

**Figure 7:**
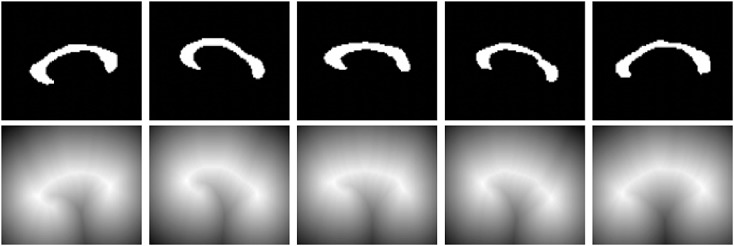
Training shapes of the corpus callosum: (top row) manually segmented images and (bottom row) corresponding signed distance functions.

The training set consists of 15 shapes of the corpus callosum, which are manually segmented from MR images. Figure 7 depicts some of the training shapes of the corpus callosum. The top row of Figure 7 depicts the manually segmented shapes, and the bottom row of Figure 7 shows the corresponding signed distance functions of the training shapes. The shape information is then incorporated to the active contour model to segment the corpus callosum structures from images of which the shapes are not included in the training set. In particular, the active contour is used to segment the corpus callosum shapes from 20 image datasets. The extracted contours are compared with manual segmentation to show the efficiency of the proposed model.

**Figure 8:**
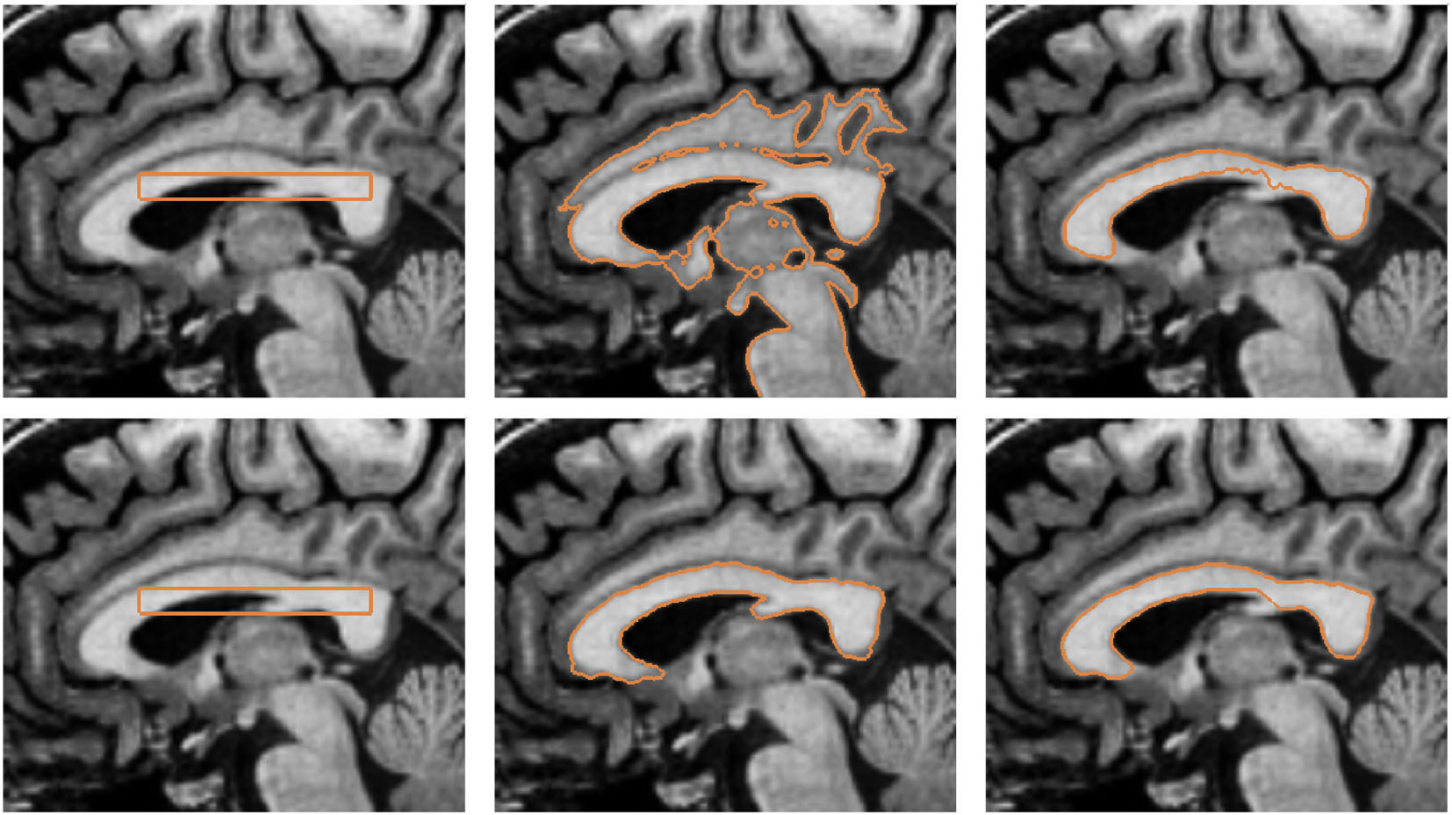
Segmentation of corpus callosum from magnetic resonance image: (top row, from left to right) initial contour, Chan-Vese model, Chan-Vese model with shape prior, (bottom row, from left to right) initial contour, gradient vector interaction-based energy, gradient vector interaction, and shape-based energy.

**Figure 9:**
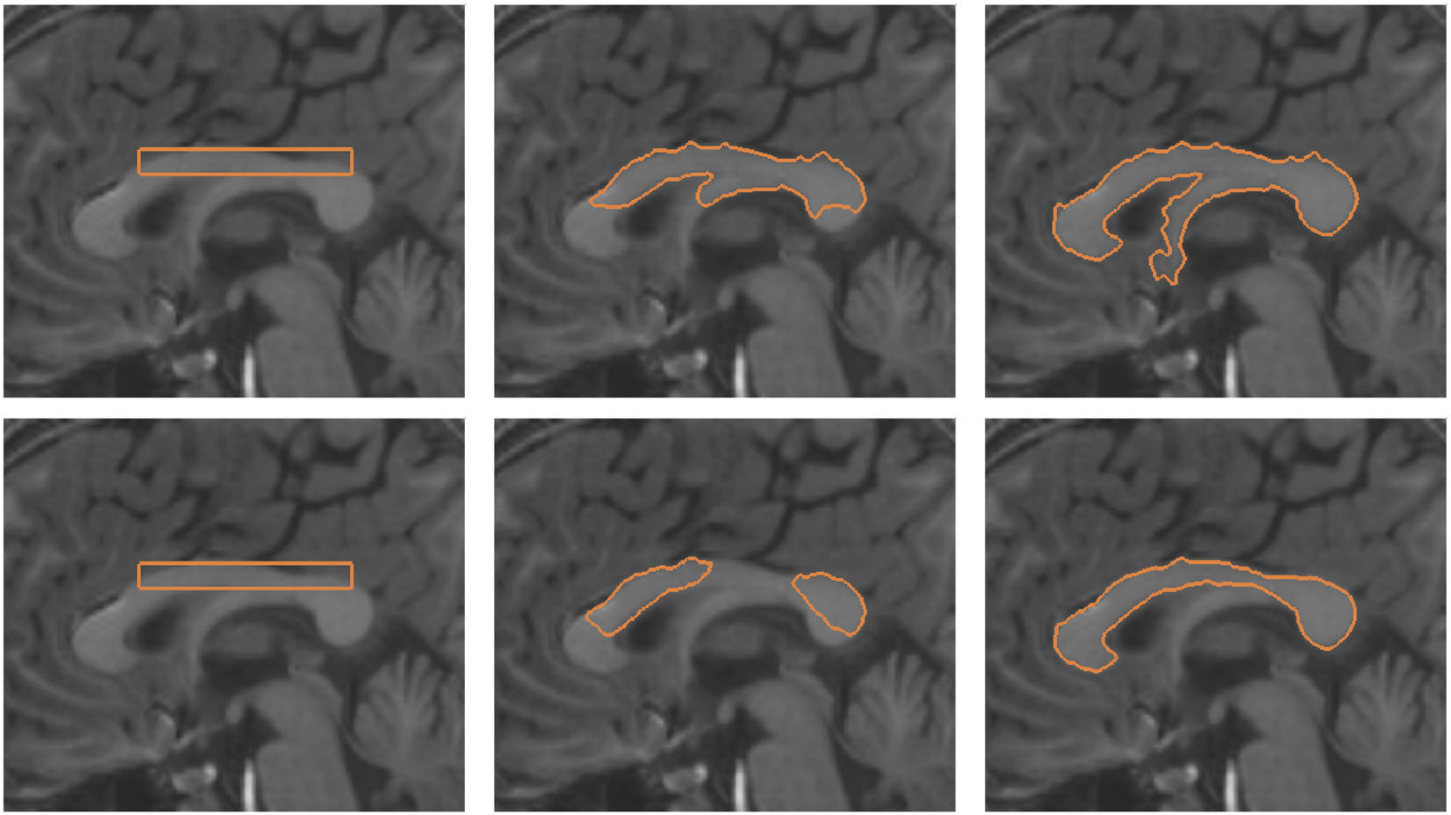
Segmentation of corpus callosum from magnetic resonance image using the proposed active contour: (top row) gradient vector interaction-based energy, (bottom row) gradient vector interaction, and shape-based energy.

**Figure 10:**
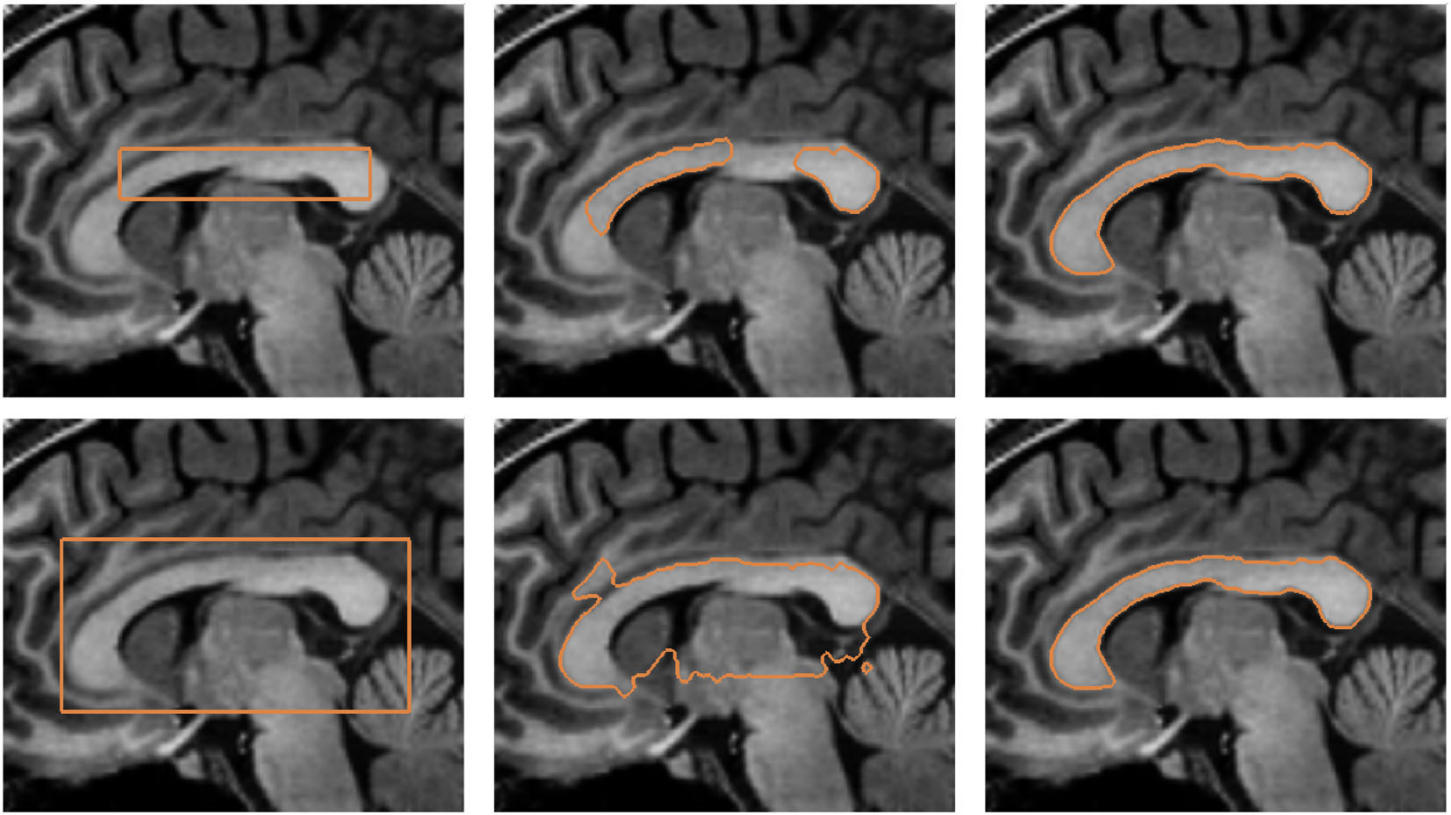
Segmentation of corpus callosum from magnetic resonance image using the proposed active contour with shape prior using different initializations: (top row) initialization across boundary of object, (bottom row) initialization outside boundary of object.

Figure 8 depicts the segmentation of the corpus callosum from MR image. The top row of the figure shows the initial contour and converged contour of the Chan-Vese model and Chan-Vese model with shape-based energy. The bottom row of the figure depicts the initial contour and converged contour using the gradient vector interaction-based energy, and the proposed model with shape prior. It is shown that the gradient vector interaction model is more efficient than the region-based Chan-Vese model in segmenting the brain image, as the Chan-Vese model leaks out to various structures in the image. The proposed model with shape prior also provides a more accurate segmentation than the region-based model with shape prior. Figure 9 shows another example in which the proposed method is applied to segment the corpus callosum. As shown in the figure, the active contour, which uses only the image-based energy, leaks out to include the fornix structure because of the similar intensity. In contrast, the proposed model with shape prior efficiently segment the shape from the image. In Figure 10, it is shown that the proposed active contour with shape prior is highly invariant to initializations as the active contour converged accurately to the object shape using different initializations. As shown in the figure, the active contour is initialized across different structures in the image. This makes it difficult for techniques that uses only image information to extract the geometry, as image forces generated in other structures may cause the active contour to converge to various structures with similar intensity. It is shown that the proposed active contour with shape prior can effectively overcome the image-based force generated in other structures to converge to the geometry of the corpus callosum.

**Figure 11:**
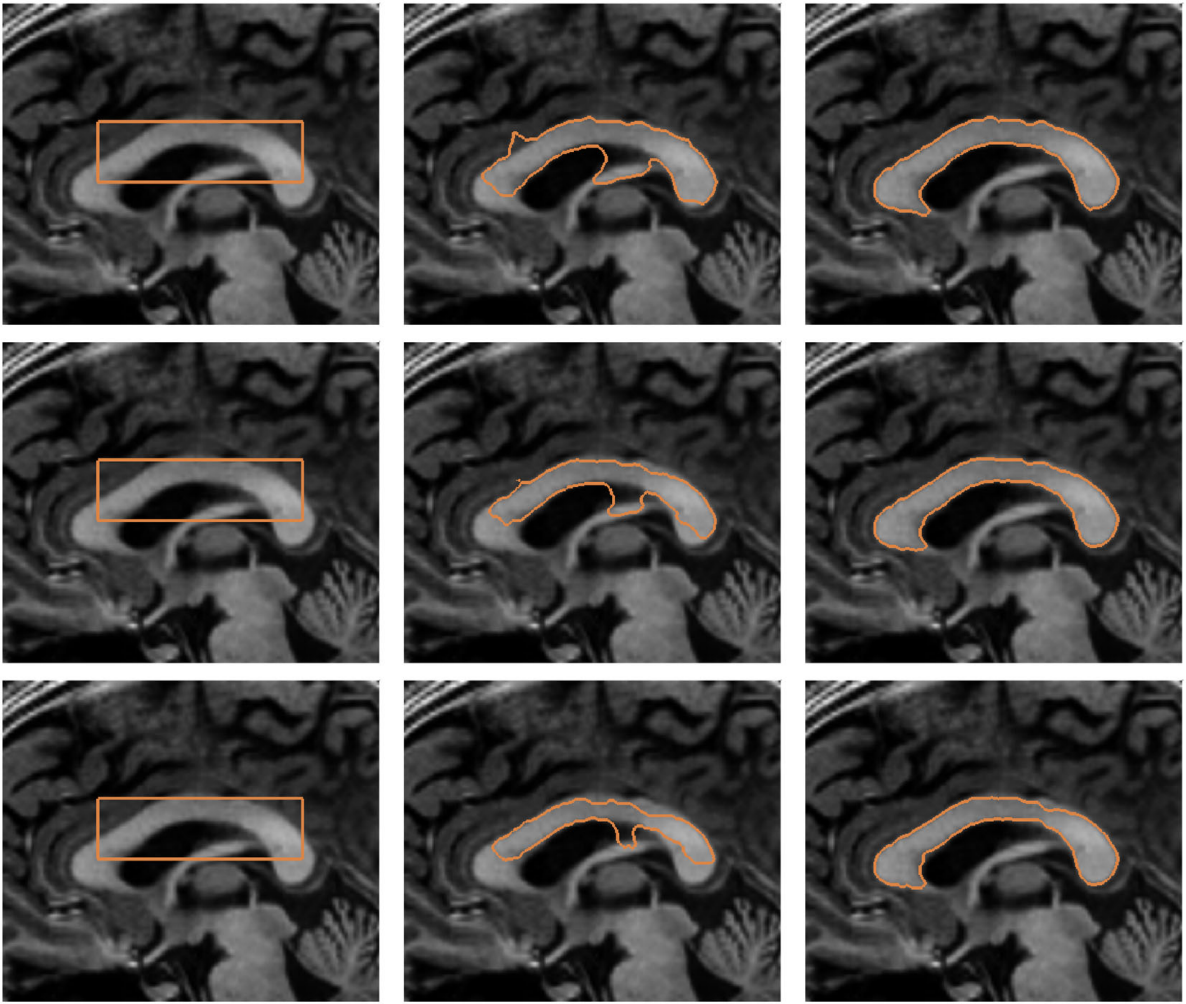
Segmentation of corpus callosum from magnetic resonance image using the proposed active contour with shape prior using different parameters: (top row) *ν* = 2.0 × 10^4^, (middle row) *ν* = 3.5 × 10^4^, and (bottom row) *ν* = 5.0 × 10^4^.

As discussed earlier that the setting of *ν* is important and often application dependent. However, the proposed method has a good tolerance to parameter settings. Figure 11 depicts the segmentation of the corpus callosum using the proposed model with different values of the weighting parameter *ν*. It is shown that the active contour with different weighting parameters, *ν* = 2.0 × 10^4^, *ν* = 3.5 × 10^4^, and *ν* = 5.0 × 10^4^, converged to the shape of the corpus callosum to segment the image structure accurately.

**Figure 12:**
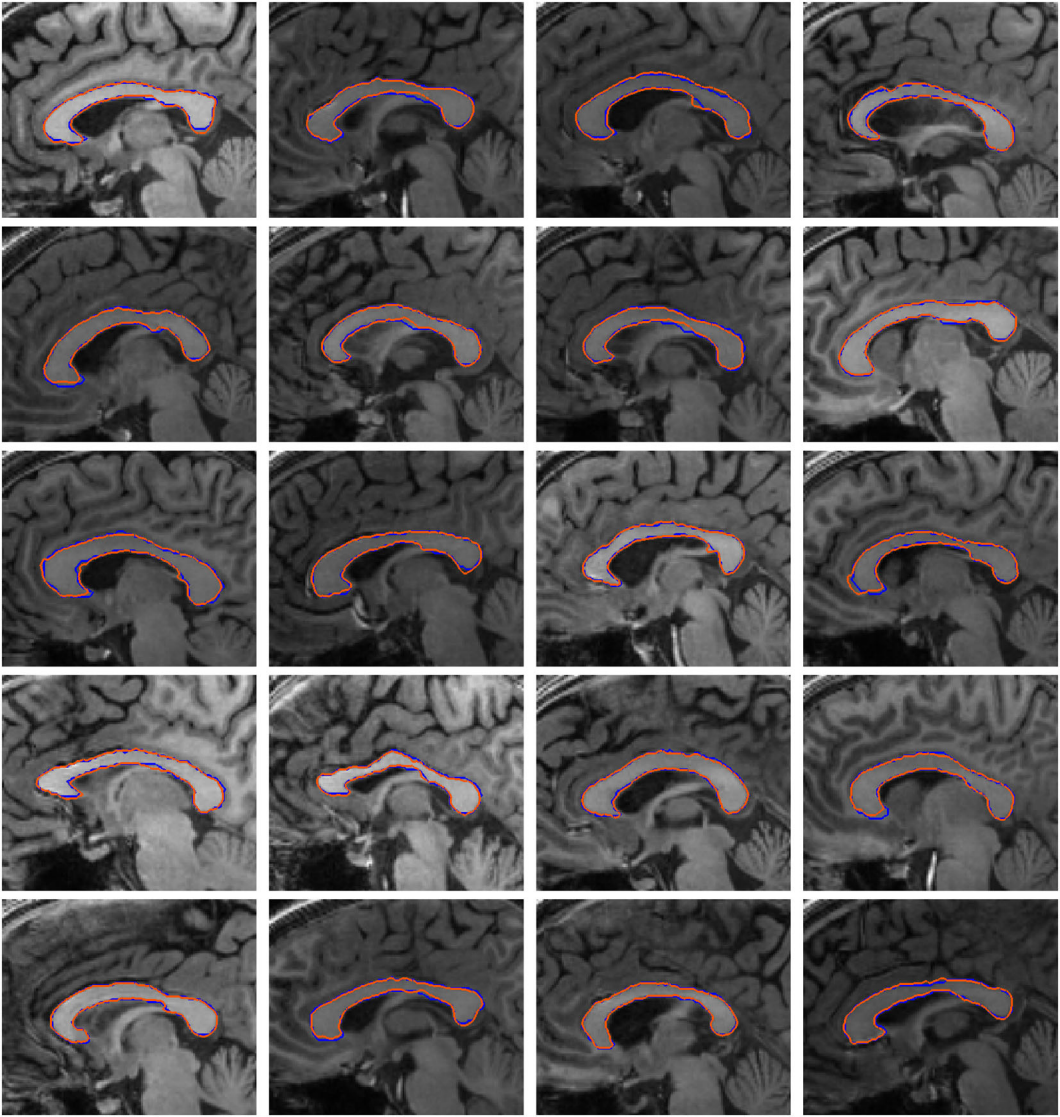
Comparison of contours extracted using the proposed active contour and manual segmentation: blue–manual, orange–proposed active contour.

Figure 12 depicts the comparison of the extracted contours using the proposed model with shape prior and manual segmentation. The blue contours represent the shapes extracted manually, and the orange contours represent the shapes extracted using the proposed active contour with shape prior. It is shown that the shapes segmented using the proposed model coincides closely with the manually extracted shapes. Table 1 presents the accuracy of the extracted contours using the proposed active contour with shape prior. The foreground (FG) and background (BG) accuracy of the extracted shapes are defined as the percentages of true FG and BG pixels, which are classified as FG and BG, respectively. A normalized overall accuracy given as the average of FG and BG is used to measure the accuracy of correctly extracted pixels, so as to reduce the amount of measurement bias toward the large number of BG pixels. It is shown that the proposed model provides accurate segmentation of the corpus callosum structures with an average FG, BG, and overall accuracy of 95.7%, 99.7%, and 97.7%, respectively.

**Table 1:** Comparison of the segmented corpus callosum geometries using the proposed model with manual segmentation: Foreground (FG), background (BG), and overall accuracy measured in %.

Dataset	Accuracy(%)		Dataset	Accuracy(%)	
	FG	97.2		FG	99.4
1	BG	99.5	11	BG	99.5
	Overall	**98.3**		Overall	**99.5**
	FG	92.3		FG	96.5
2	BG	99.9	12	BG	99.5
	Overall	**96.1**		Overall	**98.0**
	FG	98.4		FG	98.4
3	BG	99.3	13	BG	99.5
	Overall	**98.9**		Overall	**98.9**
	FG	97.9		FG	93.6
4	BG	99.5	14	BG	99.8
	Overall	**98.7**		Overall	**96.7**
	FG	94.1		FG	95.4
5	BG	99.8	15	BG	99.8
	Overall	**96.9**		Overall	**97.6**
	FG	96.4		FG	93.6
6	BG	99.8	16	BG	99.9
	Overall	**98.1**		Overall	**96.7**
	FG	89.6		FG	97.5
7	BG	99.8	17	BG	99.8
	Overall	**94.7**		Overall	**98.6**
	FG	96.0		FG	90.2
8	BG	99.8	18	BG	99.9
	Overall	**97.9**		Overall	**95.1**
	FG	95.6		FG	97.4
9	BG	99.4	19	BG	99.9
	Overall	**97.5**		Overall	**98.6**
	FG	98.9		FG	96.1
10	BG	99.6	20	BG	99.7
	Overall	**99.3**		Overall	**97.9**
**FG Average (%)**				**95.7**	
**BG Average (%)**				**99.7**	
**Overall Average (%)**				**97.7**	

**Figure 13:**
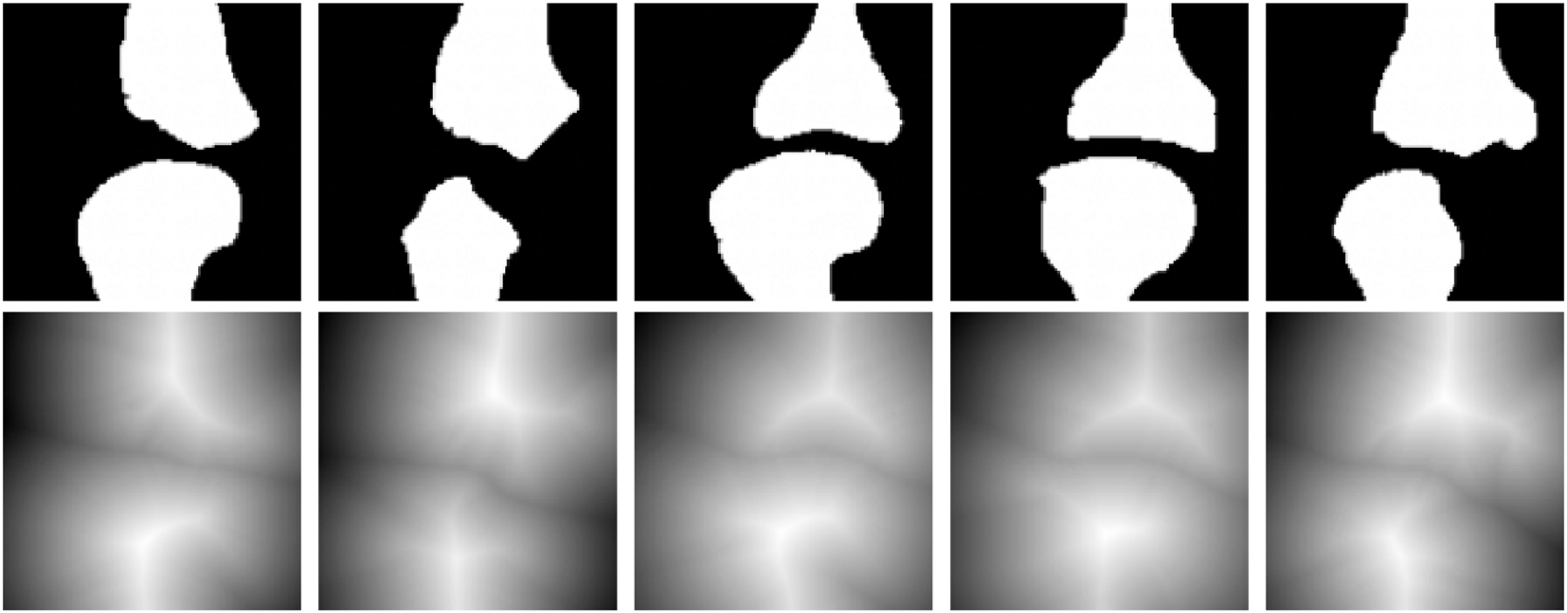
Training shapes of the knee: (top row) manually segmented images and (bottom row) corresponding signed distance functions.

The proposed model is also applied in the segmentation of the knee from MR image. The training set consists of 15 shapes manually segmented from the MR image dataset. Figure 13 depicts some of the shapes in the training set. The top row of Figure 13 shows the shapes segmented manually from the MR images, and the bottom row of Figure 13 shows the corresponding signed distance functions of the training shapes.

**Figure 14:**
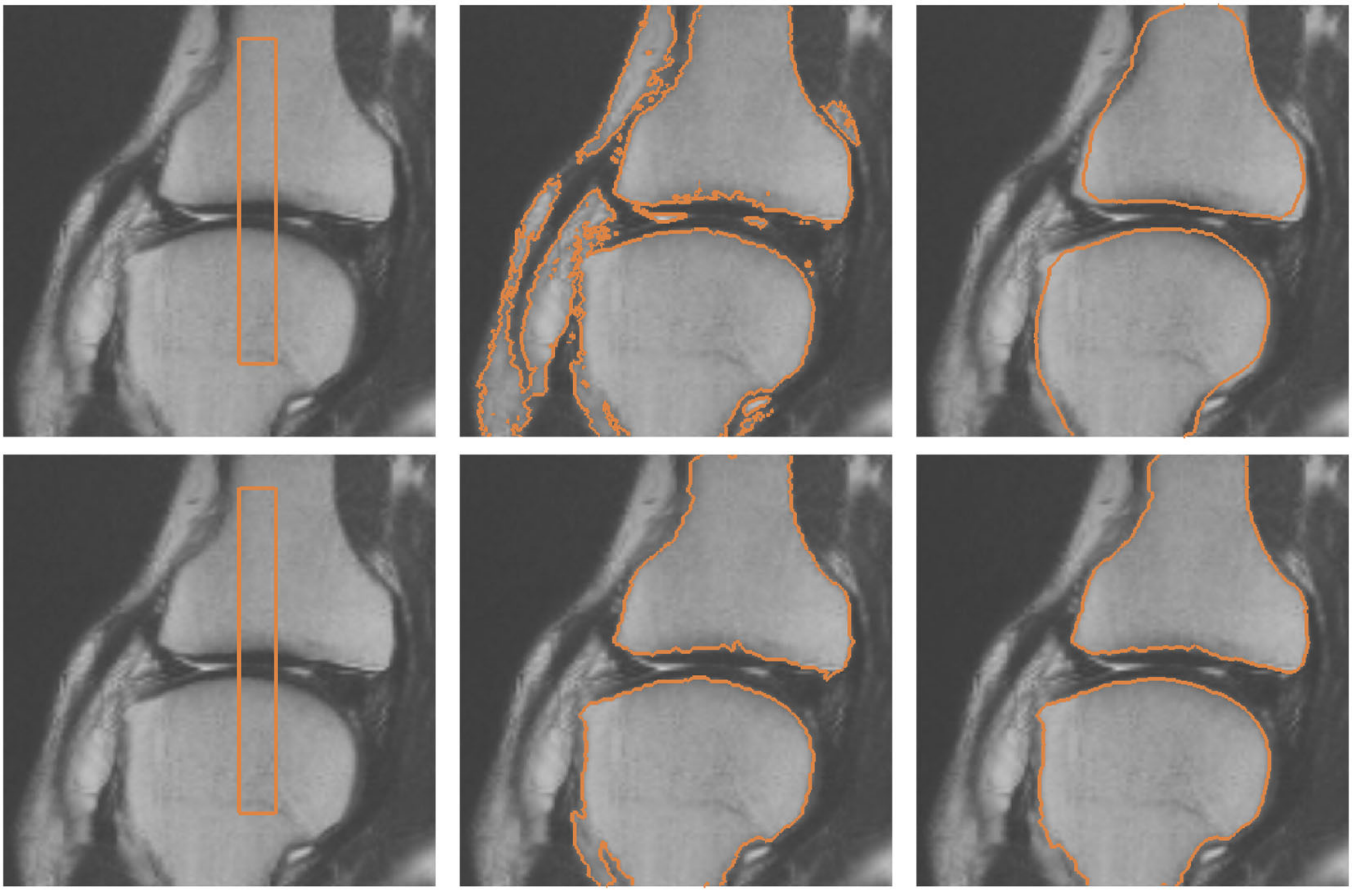
Segmentation of the knee from magnetic resonance image: (top row, from left to right) initial contour, Chan-Vese model, Chan-Vese model with shape prior, (bottom row, from left to right) initial contour, gradient vector interaction-based energy, gradient vector interaction, and shape-based energy.

**Figure 15:**
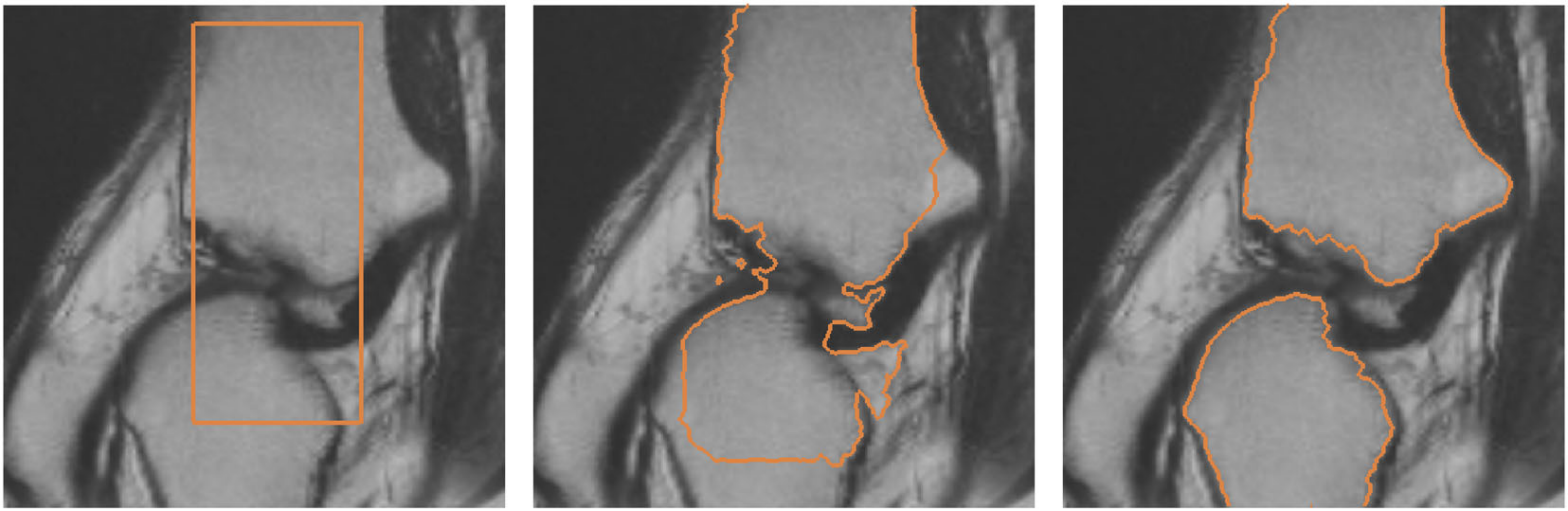
Segmentation of the knee from magnetic resonance image using gradient vector interaction-based and shape-based energies.

Figure 14 depicts the segmentation of the knee using various active contour models. The top row of the figure shows the segmentation of the knee using the Chan-Vese model and Chan-Vese model with shape prior. The bottom row of the figure shows the segmentation of the knee using the gradient vector interaction-based energy and proposed model with gradient vector interaction-based and shape-based energies. It is shown that the gradient vector interaction-based energy provides a more robust segmentation of the knee as compared with the region-based energy. The incorporated shape prior information also gives a more accurate segmentation with the proposed method. Figure 15 shows another example in which the proposed model with shape prior is used to segment the knee from the image. As shown in the figure, the global shape-based energy attracts the active contour to similar shapes in the training set, while the gradient interaction-based energy allows the active contour to locate the object boundaries to segment the shapes efficiently.

**Figure 16:**
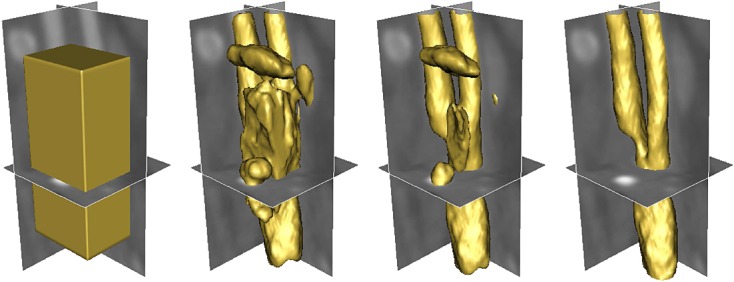
Segmentation of the carotid from computed tomography image using gradient vector interaction-based and shape-based energies.

Figure 16 depicts the segmentation of the carotid geometry from computed tomography image using the proposed model. In this example, 20 training shapes are manually generated to model the shape distribution. Note that the image data consists of various structures such as adjacent vessels and bones, and image regions representing the carotid may often contain diffused edges and intensity inhomogeneity. Therefore, careful initializations are often required for purely image-based segmentation model to extract the shape of the structure. In contrast, given an arbitrary initialization across various structures in the image as shown in Figure 16, the proposed deformable model with shape prior can overcome the image-based forces generated by other image structures to segment the carotid geometry efficiently.

## 4. Conclusion

A new variational model for level set segmentation with statistical shape prior has been presented. The image-based energy derived from the global interaction of gradient vectors provides a more coherent and global representation of the geometric configuration. The active contour model is thus more robust to image noise and weak edges, and has a strong invariance to initializations. By using kernel density estimation, the incorporated shape prior can model arbitrary shape distributions. The proposed model can thus segment complex shapes from occluded and noisy images effectively. Several examples are provided using various object shapes from synthetic and real images. It is shown that the proposed model with gradient vector interaction-based and shape-based energies can be used to segment various object shapes from biomedical images efficiently.
